# Shop until you drop: alternative interpretations

**DOI:** 10.1007/s12471-021-01549-8

**Published:** 2021-02-22

**Authors:** A. P. M. Gorgels, A. A. Wilde

**Affiliations:** 1 Department of Cardiology, Maastricht University Medical Centre+, Cardiology Centres of the Netherlands, location Utrecht, Utrecht, The Netherlands; 2 Department of Cardiology, Amsterdam UMC, Amsterdam, The Netherlands

Dear editor,

Baris and van den Bos should be commended for describing an interesting arrhythmia [1]. However, we would like to offer alternative explanations.

Their ladder diagram comprises sinus rhythm with 2:1 atrioventricular block and a junctional escape rhythm. Our interpretation is as follows:

Group beating is present; QRS complexes after the longer pause are closely followed by a negative P wave in leads II and III (compare end of QRS complexes with those during sinus rhythm). Clear negative P waves are seen after the QRS complexes with the shorter R‑R intervals. Therefore no sinus rhythm is present, but a junctional escape rhythm (escape interval 1120 ms) with shortly coupled retrograde conduction to the atria. Antegrade conduction to the ventricles leads to a reciprocal beat with a long coupling interval (900 ms). The latter beat also shows retrograde conduction with a longer R‑P interval, due to the shorter preceding R‑R interval, but without antegrade conduction. This leads to the next junctional escape beat (1120 ms), followed by the same sequence of events (Fig. 1).

**Fig. 1:**
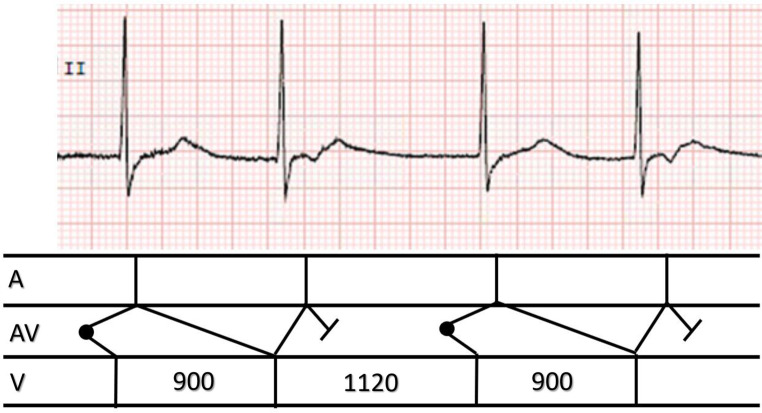
One group of repeating changes in QRS intervals and ladder diagram. See text

Another mechanism, but less likely, given the vagal state, could be: sinus arrest with an accelerated junctional rhythm with 3:2 Wenckebach exit block and retrograde conduction with longer R‑P intervals following shorter preceding R‑R intervals (Fig. 2).

**Fig. 2:**
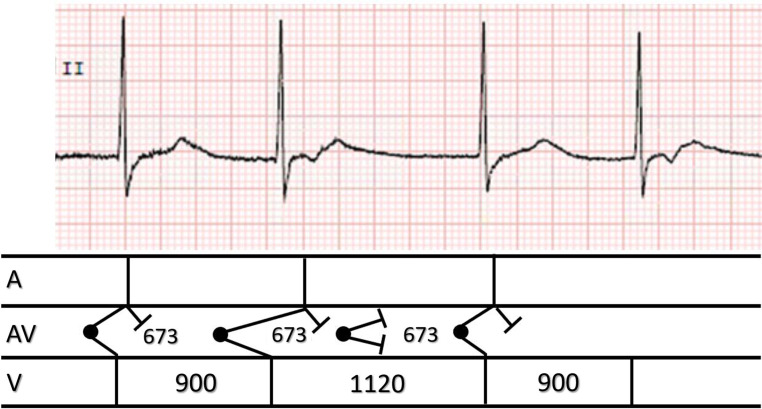
One group of repeating changes in QRS intervals and ladder diagram. See text

## References

[1] Baris L, van den Bos EJ (2020). Shop till you drop. Neth Heart J.

